# Editorial: Novel Mechanisms Involved in Urinary Bladder Control: Advances in Neural, Humoral and Local Factors Underlying Function and Disease

**DOI:** 10.3389/fphys.2020.606265

**Published:** 2020-11-10

**Authors:** Monica A. Sato, Laurival A. De Luca, Patrik Aronsson, Russ Chess-Williams

**Affiliations:** ^1^Department of Morphology and Physiology, Faculdade de Medicina do ABC, Centro Universitario Saude ABC, Santo Andre, Brazil; ^2^Department of Physiology and Pathology, Faculty of Dentistry, São Paulo State University, Araraquara, Brazil; ^3^Department of Pharmacology, Institute of Neuroscience and Physiology, University of Gothenburg, Sahlgrenska Academy, Gothenburg, Sweden; ^4^Faculty of Health Sciences & Medicine, Bond University, Gold Coast, QLD, Australia

**Keywords:** urinary bladder, neural, humoral, local factors, lower urinary tract

## Introduction

Urinary bladder (UB) dysfunctions affect the daily life activities and well-being of millions of people worldwide, leading to social and mental discomfort (Hashim et al., 2009). Lower UB disorders are not always treated effectively and their etiology is also unknown. Moreover, the increase in life expectancy due to the improvement of public health conditions globally has contributed to an increase in UB dysfunctions as a greater number of women enter menopause (Varella et al., 2016). The understanding of all such issues is crucial for better therapeutic approaches.

In spite of the increasing knowledge of effects of injuries and neurodegenerative diseases on precise traditional pontine-spinal pathways (Verstegen et al., 2017), we are still distant from a full understanding of the mechanisms involved in UB dysfunctions. We also know that stress, trauma, anxiety, or depression, including witness trauma and post-traumatic stress disorder, are behind the etiology UB pathologies (Rothrock et al., 2001; Lai et al., 2016; Bradley et al., 2017). However, there is scant information on how psychological stress affects bladder mechanisms at local tissue level. In addition, novel mechanisms involving neurohypophysial hormones highlight a role for both vasopressin and oxytocin in the control of UB smooth and detrusor muscle (Crankshaw, 1989; Pandita et al., 1998; Cafarchio et al., 2016, 2018). Finally, diabetic bladder dysfunction (DBD) is one of the most common complications of diabetes and despite the pathophysiology of DBD has been recognized as multifactorial, including alterations in the detrusor and urethra smooth muscles, urothelium, blood vessels, and autonomic nerves (Yoshiyama et al., 2015), the mechanisms underlying bladder dysfunctions in diabetic patients are not fully understood.

This current Research Topic gives a glimpse of what is currently going on in this highly interesting area, by emphasizing a role for forebrain, psychological, and humoral mechanisms in the control of micturition.

Tish and Geerling review recent phenotyping of forebrain neurons descending from the prefrontal cortex to classic micturition reflex circuitry to support a putative effect of brain injuries on the control of continence.

Cafarchio et al. show that low dose of oxytocin, which does not elicit cardiovascular effects, decreases intravesical pressure in anesthetized rats activating receptors present in the bladder, suggesting that oxytocin causes UB relaxation in lower concentration.

de Oliveira et al. show that methylglyoxal (MGO) causes bladder tissue disorganization, inflammatory infiltration in the bladder and the cystometry indicated the development of an overactive bladder profile. Indeed, those findings suggest that scavengers of MGO could be an option to ameliorate bladder dysfunction in diabetic conditions. In addition, novel drug targets for the treatment of bladder contractile dysfunctions are still necessary. Moreover, Stromberga et al. compare the responses of PGE_2_ on the bladder mucosa and detrusor muscle showing that prostaglandin receptor systems are potential regulators of UB contractility.

West et al. demonstrate in mice that social defeat reduces voiding frequency, nevertheless evoking an increased urothelial acetylcholine release during bladder distension, whereas witness trauma showed no change in voiding behavior. Thereby, the findings suggest that functional bladder changes are dependent on stressor type.

Finally yet importantly, Magaldi et al. show the effects of ovariectomy on morphological and functional changes in UB and demonstrate that resistance exercise performed chronically reverses the functional and morphological changes caused by hypoestrogenism.

Taken together, urinary bladder control is a multifaceted topic including several aspects of signal transmission and disease models. Scientific progress within this field would undoubtedly increase the quality of life for a vast number of people globally, which may be why researchers from different disciplines work to solve their “piece of the puzzle.” The advances in the understanding of the mechanisms involving neural, humoral, and local factors which underlie UB function and disease can contribute for better therapeutic models upon the increasing number of people with lower urinary tract dysfunctions worldwide.

## Author's Note

This Research Topic emphasized recent advances in psychological, humoral, and local factors involved in urinary bladder function. The understanding of novel mechanisms which control micturition is crucial for the development of new therapeutic strategies as the number of bladder dysfunctions has increased in the population worldwide.

## Author Contributions

All authors listed have made a substantial, direct and intellectual contribution to the work, and approved it for publication.

## Conflict of Interest

The authors declare that the research was conducted in the absence of any commercial or financial relationships that could be construed as a potential conflict of interest.
